# Mechanistic Insights into Pancreatic Lipase Inhibition by Sugarcane Polyphenols: A Structural and Kinetic Study

**DOI:** 10.3390/foods15091480

**Published:** 2026-04-23

**Authors:** Qiyan Liu, Ping-Ping Wang, Xiong Fu, Chun Chen

**Affiliations:** 1 School of Food Science and Engineering, South China University of Technology, Guangzhou 510640, China; 2 School of Chemical Engineering and Light Industry, Guangdong University of Technology, Guangzhou 510006, China; 3 Zhuhai Institute of Modern Industrial Innovation, South China University of Technology, Zhuhai 519175, China; 4 Overseas Expertise Introduction Center for Discipline Innovation of Food Nutrition and Human Health (111 Center), Guangzhou 510640, China

**Keywords:** sugarcane polyphenols, pancreatic lipase inhibition, mixed-type inhibition, structural perturbation, molecular docking, anti-obesity

## Abstract

Pancreatic lipase (PL) inhibition is a promising dietary strategy for obesity management. In this study, the inhibitory mechanisms and structural basis of polyphenols extracted from different sugarcane fractions were investigated using in vitro enzyme assays, spectroscopy, and molecular docking analyses. PL inhibitory activity was evaluated using p-nitrophenyl laurate (pNPL) as the substrate, with all assays performed in triplicate and results statistically analyzed. Among the extracts, sugarcane peel polyphenols (SP) exhibited the strongest inhibition, with a half-maximal inhibitory concentration (IC_50_) of 31.56 mg/mL, significantly lower than that of sugarcane juice polyphenols (SJ, 55.86 mg/mL) and sugarcane bagasse polyphenols (SB, 65.31 mg/mL). Enzyme kinetic analyses revealed a reversible mixed-type inhibition mechanism. In contrast to crude extracts, individual phenolic monomers showed substantially lower IC_50_ values (0.13–1.33 mg/mL), highlighting the intrinsic dilution. Compositional analysis identified ferulic acid, gallic acid, chlorogenic acid, and schaftoside as key contributors to PL inhibition. Fourier transform infrared (FTIR) and fluorescence spectroscopy demonstrated that polyphenols altered PL secondary structure by modulating α-helix and β-sheet contents and perturbed the microenvironment of tryptophan (Trp) and tyrosine (Tyr) residues. Molecular docking further indicated that these compounds bind within or near the substrate-binding channel via hydrogen bonding and hydrophobic interactions, engaging critical residues including Ser152, His263, and Phe77, and potentially influencing conformational elements involved in active-site accessibility. Collectively, these results suggest that sugarcane, particularly its peel, represents a valuable natural source of PL inhibitors. Despite the relatively high IC_50_ values of crude extracts, their inhibitory activity arises from multicomponent contributions and supports their potential application as dietary modulators of fat digestion rather than as pharmaceutical lipase inhibitors.

## 1. Introduction

Obesity has evolved into a global health crisis of unprecedented scale, presenting a complex and multifaceted challenge to healthcare systems worldwide. China’s rapid socioeconomic transition since the 1990s has been accompanied by a dramatic surge in overweight and obesity rates. Under current national diagnostic criteria, approximately 50% of adults and 20% of children are now classified as overweight or obese, establishing China as the country with the world’s largest obese population [1]. This escalating trend is deeply concerning given the robust epidemiological associations between obesity and a spectrum of serious non-communicable diseases, including type 2 diabetes, hypertension, cardiovascular disorders, metabolic syndrome, and several obesity-related cancers [2]. The substantial morbidity, mortality, and economic burden imposed by obesity underscore the critical need for effective, safe, and sustainable strategies for its prevention and management.

Dietary fat absorption represents a primary contributor to excessive caloric intake, with pancreatic lipase (PL) serving as the pivotal enzyme in this process. PL, secreted into the small intestine, catalyzes the hydrolysis of dietary triglycerides into absorbable monoacylglycerols and free fatty acids, constituting the principal route for lipid-derived energy acquisition [3]. As the rate-limiting step in dietary fat digestion, PL represents a well-validated pharmacological target for obesity management. The inhibition of PL activity can effectively reduce intestinal lipid hydrolysis and absorption, thereby lowering overall caloric intake. Orlistat, a synthetic derivative of lipstatin, remains the primary clinically approved anti-obesity drug acting via this mechanism. It functions as a potent, reversible inhibitor of gastrointestinal lipases, blocking approximately 30% of dietary fat absorption [4]. However, its clinical utility is significantly constrained by a high incidence of undesirable gastrointestinal adverse effects—such as steatorrhea, abdominal cramps, flatulence, and fecal urgency—which often lead to poor patient adherence and limit its broader application [5].Consequently, the identification and development of novel, naturally derived PL inhibitors with improved safety and tolerability profiles has emerged as a compelling research frontier in nutritional and preventive medicine.

In this context, plant-derived polyphenols have attracted considerable scientific interest as promising candidates for natural PL inhibition. A growing body of evidence indicates that various polyphenol-rich extracts and isolated phytochemicals exhibit significant in vitro PL-inhibitory activity. For example, tannins from pistachio green hulls demonstrate dual antioxidant and anti-lipase properties [6]. Extracts from *Phyllanthus emblica* (Indian gooseberry) show superior PL inhibition compared to those from *Eucalyptus globulus* extracts and *Tinospora sinensis* [7]. Polyphenols from onion flowers have exhibited anti-obesity and antioxidant effects in *Caenorhabditis elegans* models [8], while compounds extracted from coffee leaves have also been reported to inhibit PL [9]. These collective findings underscore the potential of dietary polyphenols to serve as foundational components in the development of functional foods or nutraceuticals aimed at modulating lipid metabolism and managing weight.

Despite extensive reports on plant-derived polyphenols as pancreatic lipase inhibitors, most existing studies primarily focus on inhibitory potency without sufficiently addressing enzyme conformational regulation. While many natural extracts show promising results, their specific modes of action remain fragmented, lacking comparative evidence across different anatomical parts of a single botanical source. Pancreatic lipase activity is known to be controlled by flexible structural regions that regulate access to the catalytic site, and modulation of these regions can significantly influence enzymatic efficiency. However, the extent to which dietary polyphenols from different plant tissues affect pancreatic lipase activity through differential kinetic behavior and structural perturbation remains poorly understood. In particular, comparative evidence linking polyphenol composition to conformational regulation of pancreatic lipase across distinct botanical fractions is still limited.

Sugarcane (*Saccharum* spp.), ranking among the world’s most vital sugar crops, holds substantial economic and agricultural importance. Beyond its primary role in sucrose production, sugarcane and its processing byproducts—such as molasses [10], bagasse [11], and peel—are increasingly recognized as valuable reservoirs of bioactive polyphenolic compounds. These compounds, including phenolic acids and flavonoids, have been associated with a range of beneficial biological activities, such as antioxidant, anti-inflammatory, antidiabetic, and hepatoprotective effects [12,13]. Despite this recognized bioactivity, significant knowledge gaps persist. Notably, the distribution, compositional profile, and relative potency of polyphenols across different anatomical parts of the sugarcane plant (e.g., peel, juice, bagasse) remain inadequately characterized and compared. More importantly, the specific inhibitory efficacy of these sugarcane-derived polyphenols against PL, the detailed kinetics of their inhibition, and the underlying molecular mechanisms—particularly how they interact with and structurally perturb the PL enzyme—are not yet systematically elucidated. Addressing these gaps is essential for transforming agricultural byproducts into high-value, health-promoting ingredients.

To bridge this knowledge deficit, the present study was designed with the following objectives: (1) to comprehensively compare the PL-inhibitory potency of polyphenolic extracts from three key sugarcane fractions: peel, juice, and bagasse; (2) to identify and quantify the major phenolic constituents responsible for the observed bioactivity; (3) to characterize the inhibition kinetics and determine the inhibition type; (4) to investigate the structural perturbations induced in PL at both secondary and tertiary levels using Fourier-transform infrared (FTIR) and fluorescence spectroscopy; and (5) to elucidate the molecular interactions and binding modes of key polyphenols with the active site of PL through integrated molecular docking and dynamics simulations. By employing this multi-technique, mechanism-driven approach, this work aims to provide a robust scientific foundation for the potential application of sugarcane-derived polyphenols, particularly from the often-discarded peel, as effective and natural functional ingredients in strategies aimed at obesity prevention and management.

## 2. Materials and Methods

### 2.1. Materials and Chemicals

Sugarcane was provided by the Guangdong Purify Health Food Co., Ltd. (Zhuhai, China). Porcine pancreatic lipase (Type II, L3126), p-nitrophenyl palmitate (pNPL), Folin–Ciocalteu reagent, gallic acid, rutin, and all phenolic acid standards (gallic, protocatechuic, chlorogenic, caffeic, ferulic, and sinapic acids) were purchased from Sigma-Aldrich (St. Louis, MO, USA). Acetonitrile and methanol (HPLC grade) were obtained from Merck (Darmstadt, Germany). All other chemicals and solvents were of analytical grade. Ultrapure water was prepared using a Milli-Q purification system (Millipore, Burlington, MA, USA).

### 2.2. Extraction of Polyphenols from Sugarcane Fractions

#### 2.2.1. Extraction from Peel and Bagasse

Polyphenols were extracted from sugarcane peel (SP) and bagasse (SB) following a modified method of Chen [14]. Briefly, the separated materials were dried at 45 °C in a forced-air oven(Shanghai Yiheng Scientific Instrument Co., Ltd., Shanghai, China), ground into a fine powder (60 mesh), and stored at –20 °C until use. Powdered samples (10 g) were mixed with 70% aqueous ethanol (1:20, *w*/*v*) and subjected to to ultrasonication using a KQ-700B benchtop ultrasonic cleaner (Kunshan Ultrasonic Instruments Co., Ltd., Kunshan, China) operated at 40 kHz and 300 W for 30 min at 25 °C. The mixture was then shaken on an orbital shaker (THZ-100, Shanghai Yetuo Technology Co., Ltd., Shanghai, China) at 150 rpm 25 °C for 12 h. After centrifugation using a centrifuge (TG16.5, Hunan Xiangyi Laboratory Instrument Development Co., Ltd., Changsha, China) at 4000× *g* for 15 min, the residue was re-extracted under identical conditions. The combined supernatants were concentrated under reduced pressure at 40 °C using a rotary evaporator (Büchi, Flawil, Switzerland) and finally lyophilized to obtain the crude polyphenol extracts. The extraction yields of sugarcane peel, juice, and bagasse polyphenols were calculated as the percentage of dried extract mass relative to the initial dry material.

#### 2.2.2. Enrichment from Sugarcane Juice

Polyphenols from fresh sugarcane juice (SJ) were enriched using AB-8 macroporous resin according to Wen et al. [15] with modifications. The juice was clarified by centrifugation and filtered. The filtrate was mixed with pre-treated AB-8 macroporous resin (S30931, Shanghai Yuanye Bio-Technology Co., Ltd., Shanghai, China) (1:10, *v*/*v*) and shaken at 180 rpm for 4 h at 25 °C to reach adsorption equilibrium. The resin was collected by filtration, washed with deionized water, and subsequently eluted with 70% ethanol (1:5, *v*/*v*) for 60 min. The eluate was concentrated and lyophilized as described above.

### 2.3. Determination of Total Polyphenol and Flavonoid Contents

Total polyphenol content (TPC) was determined using the Folin–Ciocalteu colorimetric method [16]. Briefly, an aliquot of the sample (0.1 mL) was mixed with 0.5 mL of Folin–Ciocalteu reagent (diluted 1:10 with water). After 5 min in the dark, 1.5 mL of 20% (*w*/*v*) sodium carbonate solution was added, and the volume was adjusted to 10 mL with distilled water. The mixture was incubated in the dark for 30 min, and the absorbance was measured at 763 nm using a UV-visible spectrophotometer (Shimadzu, Kyoto, Japan). TPC was calculated from a gallic acid standard curve (0–100 µg/mL) and expressed as mg gallic acid equivalents per gram of dry matter (mg GAE/g DM).

Total flavonoid content (TFC) was quantified by the aluminum chloride colorimetric method [17]. A sample aliquot (0.2 mL) was mixed with 0.8 mL of 70% ethanol, followed by sequential addition of 60 µL of 5% (*w*/*v*) NaNO_2_, 60 µL of 10% (*w*/*v*) AlCl_3_, and 0.4 mL of 1 M NaOH. The final volume was adjusted to 2 mL with distilled water. After 30 min of incubation, absorbance was read at 510 nm. TFC was determined using a rutin calibration curve and expressed as mg rutin equivalents per 100 g dry weight (mg RE/100 g DW).

TPC and TFC were determined for extract characterization and comparative analysis, rather than for dose normalization in enzyme inhibition assays.

### 2.4. Identification and Quantification of Phenolic Compounds by HPLC-DAD

Phenolic profiles of the extracts were analyzed using an Agilent 1260 HPLC system (Agilent Technologies, Santa Clara, CA, USA) equipped with a diode array detector (DAD) as previously reported [14,18]. Separation was achieved on a ZORBAX SB-C18 column (4.6 × 250 mm, 5 µm; Agilent Technologies, Santa Clara, CA, USA) maintained at 40 °C. The mobile phase consisted of 0.1% (*v*/*v*) formic acid in water (solvent A) and methanol (solvent B). The following gradient program was used at a flow rate of 0.8 mL/min: 0–11 min, 0% B; 11–12 min, 0–5% B; 12–23 min, 5–12% B; 23–35 min, 12–16.6% B; 35–50 min, 16.6–29.5% B; 50–55 min, 29.5–31% B; 55–60 min, 31–45% B; 60–70 min, 45–55% B; 70–73 min, 55% B; 73–80 min, 55–0% B. The injection volume was 20 µL. Detection wavelengths were set at 280 nm and 320 nm for phenolic acids and flavonoids, respectively.

Compounds were identified by comparing their retention times and UV–visible absorption spectra with those of commercial authentic standards (gallic acid, protocatechuic acid, chlorogenic acid, caffeic acid, ferulic acid, sinapic acid, and schaftoside; purity ≥ 98%, Sigma-Aldrich, St. Louis, MO, USA). Quantification was performed using external and internal standard calibration curves generated by plotting peak areas against six concentration levels (1–100 μg/mL) of each corresponding standard.

### 2.5. Inhibition Assay of Sugarcane Polyphenols Against Pancreatic Lipase

Extract concentrations were expressed as total extract mass (mg/mL), whereas pure phenolic monomers were tested based on compound mass concentration. PL inhibitory activity was assessed using pNPL as the substrate according to the method of Jaradat et al. [19] with modifications. The reaction mixture contained 200 µL of sample at various concentrations, 200 µL of PL solution (5 mg/mL in Tris-HCl buffer, pH 7.4), and 500 µL of Tris-HCl buffer. After pre-incubation at 37 °C for 10 min, 600 µL of pNPL solution (2.5 mM in dimethyl sulfoxide) was added to initiate the reaction. The mixture was incubated at 37 °C for 20 min, and the reaction was terminated by heating in a boiling water bath for 5 min. The amount of released p-nitrophenol was measured at 405 nm. Appropriate controls (enzyme blank, inhibitor blank, and substrate blank) were included. The inhibition percentage was calculated as follows:

$$\mathrm{Inhibition}\;(\%)\;=\;\;\left(1-\frac{A_{sample}-A_{sample\;blank}}{A_{control}-A_{control\;blank}}\right)\;\times \;100\%$$

The half-maximal inhibitory concentration (IC_50_) was determined by nonlinear regression of the inhibition curve using GraphPad Prism 9.0. Corresponding sample blanks containing extract or monomer without enzyme were included to correct for background absorbance at 405 nm. No visible precipitation or turbidity was observed under the tested concentration range during the assay period. The assay conditions were optimized for in vitro comparative inhibition analysis, rather than for direct simulation of physiological intestinal conditions.

### 2.6. Enzyme Kinetics and Inhibition Mode Analysis

Reversible inhibition and kinetic analyses of pancreatic lipase by sugarcane polyphenols were performed according to the method of Rahim ATMA et al. with slight modifications [20]. For reversible inhibition assays, sugarcane polyphenols at various concentrations were incubated with pancreatic lipase at different concentrations (3.5, 4.0, 4.5, 5.0, 5.5, and 6.0 mg/mL) using 2.5 mmol/L p-NPL as the substrate concentration.

For kinetic inhibition assays, the enzyme concentration was fixed at 5.0 mg/mL, and sugarcane polyphenols at different concentrations were reacted with varying p-NPL concentrations (1.25, 2.0, 2.5, 3.0, 3.5, and 4.0 mmol/L). Lineweaver–Burk plots were used to determine the inhibition type, and kinetic parameters were estimated using the following equation:

$$\frac{1}{V}\;=\;\frac{K_{m}}{V_{max}}\left(1+\frac{1}{K_{i}}\right)\frac{1}{\left[S\right]}\;+\;\frac{1}{V_{max}}(1+\frac{I}{K_{is}})$$

where [S] is the substrate concentration, I is the inhibitor concentration, V is the reaction velocity, $V_{max}$ is the maximum reaction velocity, $K_{m}$ is the Michaelis constant, $K_{i}\;$ and $K_{is}$ represent the inhibition constants for the free enzyme and the enzyme–substrate complex, respectively.

The apparent Michaelis constant (K_m_^app^) and apparent maximum reaction velocity (V_max_^app^) were calculated based on Lineweaver–Burk plots. Although pancreatic lipase is an interfacial enzyme, the use of the synthetic substrate p-nitrophenyl laurate (pNPL) in a DMSO/buffer system allows for a pseudo-homogeneous kinetic analysis and approximation by the Michaelis–Menten model. Therefore, the kinetic parameters obtained in this study represent apparent values suitable for comparative inhibition analysis. The assay conditions were optimized for in vitro kinetic evaluation, rather than for direct simulation of physiological intestinal conditions.

### 2.7. Fourier-Transform Infrared (FTIR) Spectroscopy

The secondary structural changes in PL induced by polyphenols were analyzed using Fourier-transform infrared spectroscopy, following the procedure reported by Li et al. with slight modifications [21]. PL solution (5 mg/mL) was mixed with an equal volume of polyphenol solution (at IC_50_ concentration) and incubated at 37 °C for 20 min. The mixture was lyophilized, and the dry powder was placed on the ATR crystal. Spectra were recorded in the range of 4000–400 cm^−1^ with a resolution of 4 cm^−1^. The amide I band (1600–1700 cm^−1^) was deconvoluted and subjected to Gaussian curve fitting using OriginPro 2022 software to quantify the proportions of α-helix, β-sheet, β-turn, and random coil structures.

### 2.8. Fluorescence Quenching Studies

The effects of sugarcane polyphenols on the tertiary structure of pancreatic lipase were evaluated using fluorescence spectroscopy, following the method described by Jiang et al. with slight modifications [22]. A pancreatic lipase solution (10 mg/mL) was pre-incubated for 5 min and then mixed with sugarcane polyphenols at various concentrations. After incubation at 298, 304, and 310 K for 5 min, fluorescence emission spectra were recorded using a fluorescence spectrometer. The excitation wavelength was set at 280 nm, and emission spectra were collected in the range of 300–500 nm. The excitation and emission slit widths were set at 5 nm and 10 nm, respectively.

The fluorescence quenching mechanisms were analyzed using the Stern–Volmer equation:

$$\frac{F_{0}}{F}\;=\;1+\;K_{sv}\left[Q\right]\;=\;I\;+\;K_{q}\tau_{0}\left[Q\right]$$

where [Q] is the concentration of sugarcane polyphenols, $F_{0}$ and F are the fluorescence intensities of pancreatic lipase in the absence and presence of polyphenols, respectively, $K_{sv}$ is the Stern–Volmer quenching constant, $K_{q}$ is the quenching rate constant, and $\tau_{0}$ is the fluorescence lifetime of the free enzyme (10^−8^ s).

The binding constant, $K_{a}$ and the number of binding sites (*n*) were calculated using the following double-logarithmic equation:

(1) $$log\frac{{(F}_{0}-F)}{F}\;=\;lgK_{a}\;+\;nlg\left[Q\right]$$

### 2.9. Molecular Docking

The crystal structure of human pancreatic lipase (PDB ID: 1LPB) was obtained from the Protein Data Bank (https://www.rcsb.org/, accessed on 15 March 2025). The three-dimensional structures of gallic acid, chlorogenic acid, caffeic acid, ferulic acid, schaftoside, sinapic acid, and the substrate pNPL (CAS: 1956-11-2) were retrieved from the PubChem database (https://pubchem.ncbi.nlm.nih.gov/, accessed on 15 March 2025). Protein preparation was performed in AutoDock Tools (ADT 1.5.7), including the removal of crystallographic water molecules, co-crystallized ligands, and ions, followed by hydrogen addition and Kollman charge assignment. Docking simulations were executed using AutoDock 4.2 based on the Lamarckian genetic algorithm. Docking results were ranked according to binding energy, and the lowest-energy conformations were visualized using PyMOL 3.1.5.1. Two-dimensional interaction diagrams were generated using AutoDock Vina 1.1.2.

### 2.10. Statistical Analysis

All experiments were performed in triplicate, and data are presented as mean ± standard deviation (SD). Statistical analysis was performed using SPSS 26.0 software. One-way ANOVA followed by Tukey’s post hoc test was used to determine significant differences among groups (*p* < 0.05). Curve fitting and parameter calculations were performed using GraphPad Prism 9.0.

## 3. Results and Discussion

### 3.1. Total Phenolic and Flavonoid Contents in Different Sugarcane Fractions

The total phenolic content (TPC) and total flavonoid content (TFC) varied significantly (*p* < 0.05) among the three sugarcane fractions (Table 1). Sugarcane peel (SP) exhibited the highest TPC (13.52 ± 0.87 mg gallic acid equivalents per gram of dry matter (mg GAE/g DM)), followed by sugarcane juice (SJ, 4.89 ± 0.39 mg GAE/g DM) and sugarcane bagasse (SB, 1.72 ± 0.13 mg GAE/g DM). A similar distribution pattern was observed for TFC, with SP containing 2074.65 ± 224.72 mg rutin equivalents per 100 g dry weight (mg RE/100 g DW), significantly higher than that in SJ (560.79 ± 90.11 mg RE/100 g DW) and SB (77.27 ± 38.06 mg RE/100 g DW). These results align with previous studies reporting that phenolic and flavonoid compounds are predominantly concentrated in the peel of sugarcane, likely as a protective response to environmental stressors [23]. The markedly higher phenolic and flavonoid levels in SP suggest its superior potential as a source of bioactive compounds compared to juice or bagasse, supporting its utilization as an underutilized byproduct for extracting value-added phytochemicals.

### 3.2. Identification of Major Phenolic Compounds in Sugarcane Fractions

**Figure 1:**
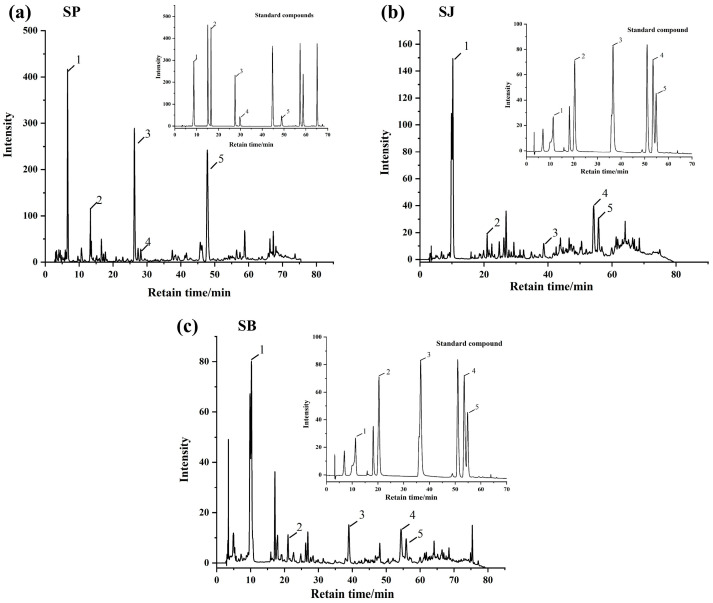
HPLC chromatograms of polyphenolic compounds in different sugarcane-derived samples and corresponding standard compounds. (**a**) Sugarcane peel (SP); (**b**) sugarcane juice (SJ); and (**c**) sugarcane bagasse (SB). In SP, the identified compounds were: (1) gallic acid, (2) protocatechuic acid, (3) chlorogenic acid, (4) caffeic acid, and (5) ferulic acid. In SJ, the identified compounds were: (1) gallic acid, (2) protocatechuic acid, (3) caffeic acid, (4) sinapic acid, and (5) schaftoside. In SB, the identified compounds were: (1) gallic acid, (2) protocatechuic acid, (3) caffeic acid, (4) sinapic acid, and (5) schaftoside. The insets show the HPLC chromatograms of the corresponding standard compounds.

HPLC-DAD analysis revealed distinct phenolic profiles across the three fractions (Figure 1 and Table 2). SP contained five phenolic acids, with ferulic acid (128.22 µg/g) and gallic acid (97.12 µg/g) as the most abundant constituents. Chlorogenic acid and ferulic acid were detected exclusively in SP. SJ and SB shared several compounds, including gallic acid, protocatechuic acid, caffeic acid, ferulic acid, and the flavonoid schaftoside. Notably, schaftoside and sinapic acid were absent in SP but present in SJ and SB. These compositional differences suggest tissue-specific biosynthesis and accumulation of polyphenols in sugarcane, which may be influenced by physiological functions, developmental stage, and environmental factors [24]. The abundance of ferulic acid and gallic acid in SP correlates with its higher TPC and TFC, and these compounds are known for their strong antioxidant and enzyme inhibitory activities, hinting at their potential role in PL inhibition.

It should be noted that the phenolic compounds listed in Table 2 represent the major constituents identified based on available authentic standards. Although additional minor peaks were observed in the HPLC chromatograms, they were not included due to the lack of reliable identification. The compounds selected for further inhibition assays were therefore used as representative phenolics to elucidate the contribution of sugarcane polyphenols to pancreatic lipase inhibition.

The functional implications of these compositional differences, particularly their relationship with pancreatic lipase inhibitory potency and multicomponent effects, are further discussed in Section 3.3.

### 3.3. Inhibitory Effects of Sugarcane Extracts and Phenolic Monomers on Pancreatic Lipase

This section aims to integrate phenolic composition with inhibitory performance to provide a mechanistic interpretation of the pancreatic lipase inhibition observed for different sugarcane fractions.

The PL inhibitory activities of the three extracts were evaluated and expressed as IC_50_ values (Table 3). SP extract demonstrated the strongest inhibition (IC_50_ = 31.56 ± 1.62 mg/mL), followed by SJ (55.86 ± 2.10 mg/mL) and SB (65.31 ± 3.19 mg/mL). This order of inhibitory potency corresponds directly with their TPC and TFC rankings, suggesting that the overall phenolic content, particularly in the peel, is a key determinant of anti-lipase activity.

To further elucidate the contribution of individual compounds, six major phenolic monomers were screened for PL inhibition (Table 3). Schaftoside exhibited the strongest inhibitory effect (lowest IC_50_), followed by ferulic acid, chlorogenic acid, and caffeic acid. In contrast, sinapic acid showed the weakest inhibition.

The superior inhibitory activity of SF may be attributed to its unique molecular configuration. Previous studies have highlighted that C-glycosylation on the A-ring of flavonoids is a critical determinant of PL inhibition. Specifically, the inhibitory activity of non-glycosylated luteolin is reported to be significantly lower than that of its C-glycosylated derivatives, underscoring the indispensable role of the C-glycosyl moiety in enhancing the enzymatic inhibitory efficacy of the luteolin skeleton [25].

Notably, the IC_50_ values of the sugarcane extracts (31.56–65.31 mg/mL) were markedly higher than those of the individual phenolic monomers (0.13–1.33 mg/mL), indicating a lower apparent inhibitory potency for the crude mixtures. This discrepancy is expected and can be rationalized by the intrinsic dilution effect of crude extract systems. The IC_50_ values of the extracts are expressed as total extract mass concentration (mg/mL), whereas the IC_50_ values of pure phenolic compounds reflect the effective concentration of the active molecules themselves. Crude plant extracts are typically complex multicomponent systems composed of phenolic and non-phenolic constituents. As summarized in a recent comprehensive review, the inhibitory potency of whole extracts is often markedly lower than that of isolated individual polyphenols [26], suggesting that interactions among coexisting components may modulate the overall enzyme–inhibitor interaction behavior.

**Figure 2:**
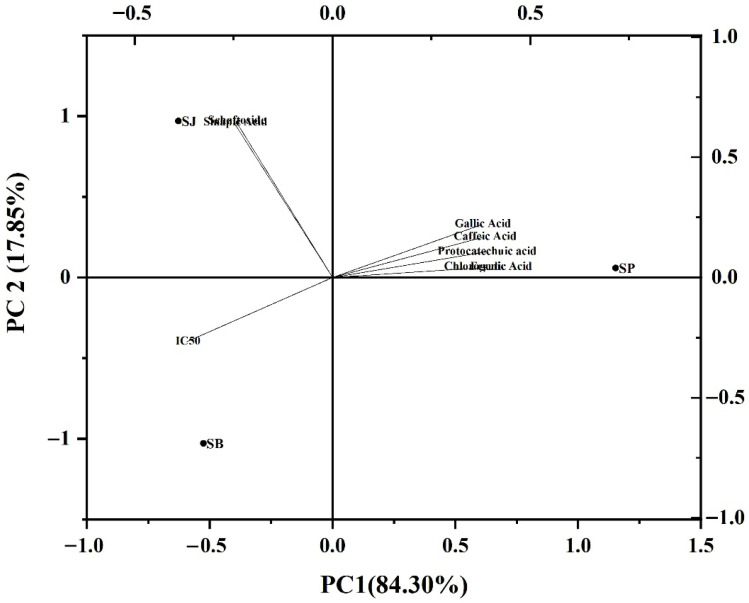
Principal component analysis (PCA) biplot of sugarcane-derived polyphenol composition and pancreatic lipase inhibition. The biplot shows the distribution of polyphenol monomers, the IC_50_ variable, and sugarcane peel (SP), sugarcane juice (SJ), and sugarcane bagasse (SB) extracts in the principal component space. Arrows represent the loadings of individual polyphenolic compounds and IC_50_, whereas dots indicate the positions of the samples. The percentages in parentheses for PC1 and PC2 indicate the variance explained by each principal component. SP was positively associated with ferulic acid, gallic acid, chlorogenic acid, caffeic acid, and protocatechuic acid, and was oriented opposite to the IC_50_ vector, indicating a negative relationship with IC_50_ and stronger inhibitory activity. In contrast, SJ and SB were distributed closer to sinapic acid and schaftoside.

Principal component analysis (PCA) further corroborated the relationship between composition and activity (Figure 2). SP clustered closely with ferulic acid, gallic acid, and chlorogenic acid, and was positioned opposite to the IC_50_ vector, indicating a strong negative correlation between these compounds and the IC_50_ value (i.e., higher content associated with stronger inhibition). In contrast, SJ and SB were located closer to the IC_50_ vector, corresponding to their lower inhibitory potency and distinct compositional profiles.

Notably, although schaftoside exhibited the strongest inhibitory activity as an individual monomer, its contribution in the PCA model was less pronounced. This apparent discrepancy can be attributed to the fact that PCA reflects the relative contribution of compounds within the complex extract matrix, which is strongly influenced by both compound abundance and activity, rather than inhibitory potency alone. Consequently, the overall inhibitory performance of the extracts is driven primarily by the combined effects of moderately active but higher-abundance phenolic acids, such as ferulic acid and chlorogenic acid, rather than by a single highly potent but less abundant constituent.

These PCA results are therefore in good agreement with the IC_50_ analysis, further supporting the notion that the inhibitory behavior of sugarcane extracts arises from multicomponent contributions rather than dominance by an individual phenolic monomer.

### 3.4. Inhibition Kinetics of Pancreatic Lipase by Sugarcane Polyphenols

Reversibility plots (Figure S1) demonstrated that the inhibition was reversible, as reaction rates remained linearly proportional to enzyme concentration and all regression lines intercepted the origin, ruling out irreversible inactivation. Subsequently, Lineweaver–Burk plots (Figure S2) revealed that all six phenolic monomers inhibited PL in a concentration-dependent manner, with increasing slopes at higher inhibitor concentrations, indicating a decline in catalytic efficiency.

The derived kinetic parameters (Table 4) demonstrated that increasing inhibitor concentration led to a decrease in the apparent maximum velocity (V_max_^app^) and an increase in the apparent Michaelis constant (K_m_^app^) for all compounds, characteristic of mixed-type inhibition. This suggests that these polyphenols can bind to both the free enzyme and the enzyme–substrate complex, thereby interfering with substrate binding and catalysis simultaneously. For instance, schaftoside induced the most pronounced changes, with K_m_^app^ increasing from 0.0312 to 1.298 mg/mL and V_max_^app^ decreasing significantly, reflecting strong interactions with PL. In contrast, chlorogenic acid and ferulic acid induced relatively smaller perturbations. The calculated inhibition constants (K_i_ and K_is_) varied among the compounds, with schaftoside showing the lowest K_i_ (0.064 mg/mL), consistent with its highest inhibitory potency.

This mixed-type inhibition mechanism is frequently reported for plant polyphenols, which often possess multiple functional groups enabling simultaneous interactions with catalytic residues and regions distal from the active site, including surface-exposed domains involved in conformational regulation [27]. The observed kinetic behavior aligns with previous studies on chlorogenic acid derivatives [28] and other fruit polyphenols, supporting the notion that sugarcane polyphenols act through a multi-target binding mode rather than simple competitive blockade of the active site. Such mixed-type inhibition is particularly plausible for pancreatic lipase, as ligand binding at regions distal from the catalytic serine can influence the conformational dynamics of surface-exposed domains, including the lid region that regulates substrate access.

Accordingly, Lineweaver–Burk analysis was employed to qualitatively evaluate inhibition modes and relative changes in apparent kinetic parameters among different polyphenols. While these parameters do not represent absolute kinetic constants under physiological lipid–water interfacial conditions, they provide a reliable comparative framework for elucidating the inhibitory behavior of sugarcane polyphenols toward pancreatic lipase.

### 3.5. Effects of Sugarcane Polyphenols on the Secondary Structure of Pancreatic Lipase

**Figure 3:**
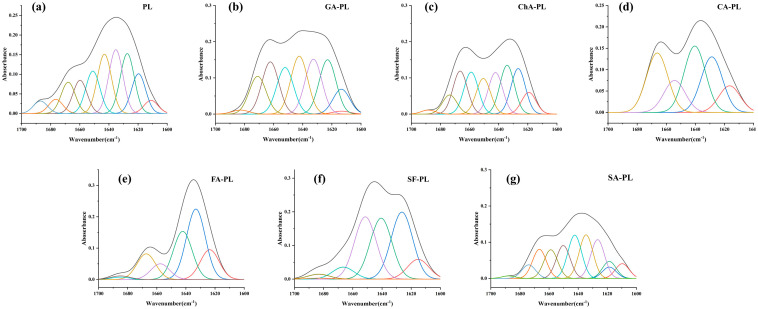
Curve fitting of the amide I region (1600–1700 cm^−1^) of pancreatic lipase (PL) and its complexes with different sugarcane polyphenols. (**a**) PL; (**b**) GA-PL; (**c**) ChA-PL; (**d**) CA-PL; (**e**) FA-PL; (**f**) SF-PL; and (**g**) SA-PL. The amide I absorption bands of blank PL and PL treated with gallic acid (GA), chlorogenic acid (ChA), caffeic acid (CA), ferulic acid (FA), schaftoside (SF), and sinapic acid (SA) were deconvoluted by Fourier self-deconvolution and Gaussian curve fitting. The black line represents the original amide I spectrum, and the colored lines represent the individual Gaussian-fitted component bands. These component bands correspond to different secondary structural elements of PL including β-sheet (1600–1640 cm^−1^), random coil (1640–1650 cm^−1^), α-helix (1650–1660 cm^−1^), and β-turn (1660–1700 cm^−1^).

ATR-FTIR spectroscopy was employed to probe conformational changes in PL upon polyphenol binding, focusing on the amide I region (1600–1700 cm^−1^). Deconvolution and curve-fitting analyses quantified the proportions of secondary structural elements (Table 5), and representative curve-fitting results of the amide I bands are shown in Figure 3. Statistical analysis revealed that the changes in α-helix and β-sheet contents induced by different polyphenols were significant (*p* < 0.05). Most phenolic acids, including gallic acid, chlorogenic acid, caffeic acid, ferulic acid, and sinapic acid, caused a significant reduction in α-helix content accompanied by an increase in random coil or β-turn structures. In contrast, schaftoside uniquely induced a significant increase in α-helix content while moderately reducing β-sheet proportion.

Notably, the lid domain of pancreatic lipase, which regulates access to the catalytic site, is known to be enriched in α-helical structures [29]. Therefore, polyphenol-induced redistribution of α-helix content may influence the conformational behavior of the lid domain and consequently affect substrate accessibility. This inference is consistent with the compound-specific inhibitory potency and kinetic behavior discussed in Section 3.3, providing indirect functional support for the observed secondary structural rearrangements.

This increase in α-helix observed for schaftoside may stabilize a less accessible conformation of the catalytic pocket, thereby hindering substrate entry or proper orientation. Similar structural shifts have been reported for PL in the presence of curcumin [30] and chlorogenic acid [31], where changes in helix content were linked to altered enzymatic activity. These findings suggest that sugarcane polyphenols can modulate the secondary structure of PL in a compound-specific manner, potentially disrupting the hydrogen-bonding network and van der Waals interactions essential for maintaining catalytic competence [32].

### 3.6. Fluorescence Quenching and Tertiary Structural Changes

**Figure 4:**
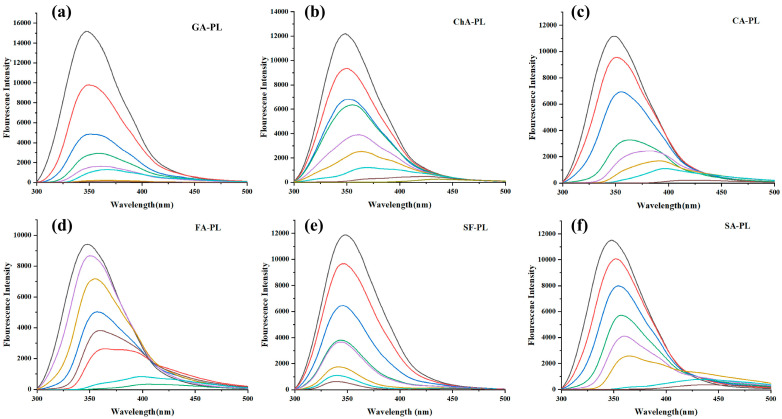
Steady-state fluorescence emission spectra of pancreatic lipase (PL) in the presence of different concentrations of sugarcane polyphenols. (**a**) GA-PL; (**b**) ChA-PL; (**c**) CA-PL; (**d**) FA-PL; (**e**) SF-PL; and (**f**) SA-PL. The excitation wavelength was 280 nm and the emission spectra were recorded from 300 to 500 nm. The black line represents PL in the absence of polyphenols, while the colored lines represent PL in the presence of increasing concentrations of the corresponding polyphenol. The fluorescence intensity of PL decreased progressively with increasing polyphenol concentrations.

Steady-state fluorescence spectroscopy was used to monitor changes in the tertiary structure of PL upon interaction with polyphenols (Figure 4). Under excitation at 280 nm, native PL exhibited a major emission peak at approximately 350 nm, and no apparent autofluorescence from the six polyphenols was observed within 300–500 nm, avoiding spectral interference. Increasing concentrations of phenolics resulted in a concentration-dependent quenching of PL intrinsic fluorescence (primarily arising from Trp and Tyr residues), accompanied by shifts in emission maxima. Red shifts were observed for GA, ChA, CfA, FA, and SA, whereas SF induced a slight blue shift. These peak-shift tendencies were consistent with structural classifications, as flavonol-3-O-glycosides were reported to induce blue shifts [33], while phenolic acids commonly caused red shifts [34].

**Figure 5:**
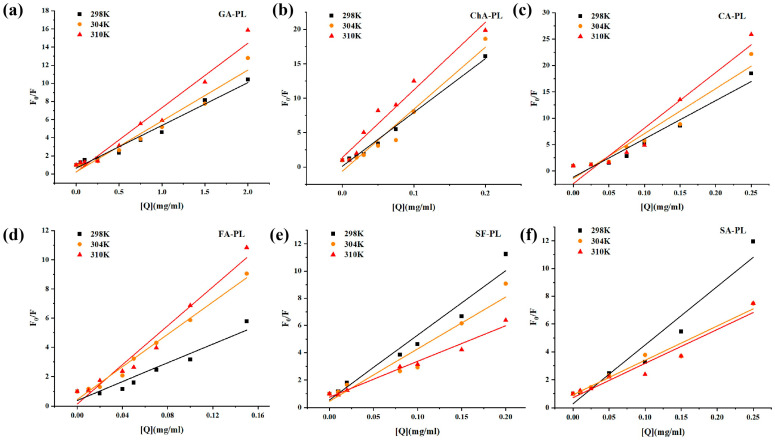
Stern–Volmer plots of pancreatic lipase (PL) in the presence of different concentrations of sugarcane polyphenols at different temperatures. (**a**) GA-PL; (**b**) ChA-PL; (**c**) CA-PL; (**d**) FA-PL; (**e**) SF-PL; and (**f**) SA-PL. Black, orange, and red lines and symbols represent the data obtained at 298, 304, and 310 K, respectively. The symbols indicate the experimental values, and the solid lines represent the corresponding linear fittings.

The Stern–Volmer plots and the corresponding fluorescence quenching parameters are summarized in Figure 5 and Table 6, respectively. Fluorescence quenching is a sensitive technique for investigating the binding interactions between small molecules and proteins. The intrinsic fluorescence of porcine pancreatic lipase is primarily attributed to tryptophan (Trp) and tyrosine (Tyr) residues. Upon the incremental addition of polyphenols, the intrinsic fluorescence intensity of PPL was significantly quenched, indicating that the microenvironment surrounding the Trp/Tyr residues in the lipase had been altered [35].

Stern–Volmer fitting (F_0_/F = 1 + Ksv[Q]) yielded good linearity (R ≈ 0.92–0.99) for all phenolics, indicating that a single Stern–Volmer model adequately described the quenching behavior. Distinct temperature-dependent trends were observed: Ksv increased with temperature for GA, ChA, CfA, and FA (e.g., ChA from 78.16 mL·mg^−1^ at 298 K to 98.25 mL·mg^−1^ at 310 K), suggesting dynamic quenching, whereas SF and SA exhibited decreasing Ksv with temperature, consistent with static quenching and ground-state complex formation.

Notably, binding constants (Ka) and stoichiometric coefficients (*n*) determined by the double-logarithmic model showed that *n* ≈ 1 (0.89–1.23) for all phenolics, implying a single dominant binding site on PL. Binding affinities varied markedly among structures, following the sequence (298 K): ChA > CfA > SF ≈ SA > FA ≫ GA. Moreover, Ka increased with temperature for ChA, CfA, and FA, whereas SF and SA displayed the opposite trend, reinforcing the dynamic–static dichotomy among structural classes.

Collectively, these spectral shifts corroborate the FTIR findings and collectively demonstrate that sugarcane polyphenols perturb the tertiary structure of PL in a structure- and mechanism-dependent manner, likely affecting the geometry and chemical environment of the active site.

### 3.7. Molecular Docking Analysis

Molecular docking simulations were conducted to elucidate the binding interactions between key sugarcane polyphenols and the active site of pancreatic lipase (PL). The predicted binding energies (ΔG) for the six phenolic compounds are summarized in Table 7. All ligands exhibited negative binding energies, indicating spontaneous binding to PL. Schaftoside (SF) showed the strongest predicted affinity (ΔG = −9.0 kJ·mol^−1^), followed by chlorogenic acid (ChA, −8.7 kJ·mol^−1^), sinapic acid (SA, −7.0 kJ·mol^−1^), ferulic acid (FA, −6.7 kJ·mol^−1^), caffeic acid (CA, −6.6 kJ·mol^−1^), and gallic acid (GA, −6.0 kJ·mol^−1^). These computational results are generally consistent with the experimental inhibition potency observed in the enzyme assays, supporting the reliability of the docking approach [36].

**Figure 6:**
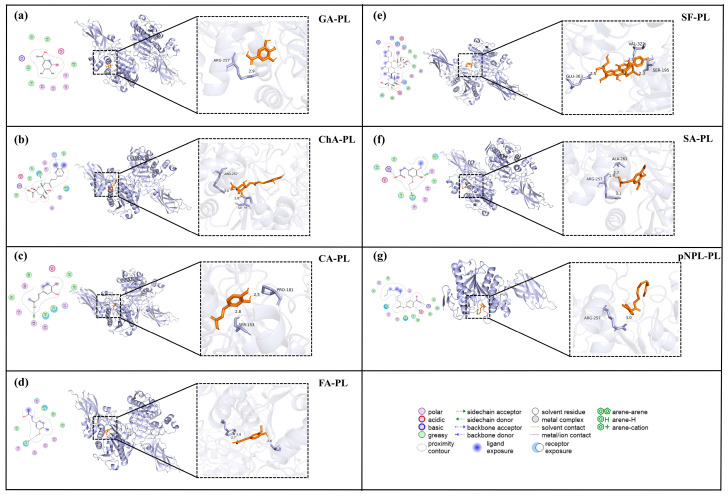
Schematic representation of the binding interactions between sugarcane-derived polyphenols and pancreatic lipase (PL). The molecular docking poses of gallic acid (GA), chlorogenic acid (ChA), caffeic acid (CA), sinapic acid (SA), schaftoside (SF), and ferulic acid (FA) with PL are illustrated, with pNPL included as a reference substrate. PL is shown as a light-blue cartoon/surface model, whereas the bound ligands are shown as stick models. The dashed boxes and arrows indicate the enlarged binding regions. The enlarged panels on the right highlight ligand–residue contacts within the PL substrate channel, including hydrogen-bond distances. Different colors and patterns in the interaction diagrams represent different types of intermolecular interactions, as defined in the legend at the bottom of the figure.

Analysis of the binding poses revealed that the polyphenols occupied distinct regions within or near the substrate-binding channel of PL (Figure 6). GA and SA primarily interacted with residues at the channel entrance, such as Arg257, Ala261, and Tyr115. FA and CA extended further into the mid-channel region, engaging residues including Phe78, Ile79, and Asp80 [37]. ChA spanned both the entrance and mid-channel areas, while SF displayed the most extensive interactions, forming contacts with multiple residues across several regions, such as Phe78, Ile79, Asp80, Ser153, Arg257, Val322, and Glu303. In comparison, the substrate pNPL adopted a pose typical of substrate recognition, with its ester bond oriented toward the catalytic Ser152 and His263, and its aromatic moiety stacked against Phe77.

The docking results further highlighted the diversity of molecular interactions governing polyphenol–PL binding. Hydrogen bonding, hydrophobic contacts, and π–π stacking were commonly observed. SF formed multiple hydrogen bonds with Asp80, Ser153, and Glu303, in addition to hydrophobic interactions with Phe78 and Ile79. ChA, FA, and CA exhibited mixed interaction profiles, combining hydrogen bonds with π-stacking. In contrast, GA and SA predominantly formed hydrogen bonds with polar residues at the channel entrance. For pNPL, key interactions included aromatic stacking with Phe78 and Tyr115, along with hydrophobic contacts along the alkyl chain.

Collectively, these docking simulations provide structural insights into the inhibitory mechanism of sugarcane polyphenols. The variation in binding location, affinity, and interaction type among the compounds correlates with their differential inhibitory activities observed in kinetics and spectroscopy studies. The ability of these polyphenols to bind within or near the active site, engaging residues critical for substrate recognition and catalysis (e.g., Ser152, His263, Phe77), supports their role as mixed-type inhibitors that interfere with both substrate access and enzyme–substrate complex formation.

## 4. Conclusions

In summary, this study systematically investigated the inhibitory behavior and mechanistic basis of polyphenols derived from different sugarcane fractions against pancreatic lipase. Among the tested extracts, sugarcane peel exhibited the strongest inhibitory activity, which was consistent with its higher phenolic content and enrichment in ferulic acid, gallic acid, and chlorogenic acid. Enzyme kinetic analysis indicated that the selected polyphenols act as reversible mixed-type inhibitors of pancreatic lipase.

Spectroscopic analyses revealed that polyphenol binding induced compound-dependent perturbations in both the secondary and tertiary structures of the enzyme, including alterations in α-helix/β-sheet proportions and changes in the microenvironment of Trp/Tyr residues. Molecular docking further supported these observations by demonstrating preferential binding of polyphenols near the substrate-binding channel through hydrogen bonding and hydrophobic interactions. Collectively, these results provide integrated biochemical and structural evidence for the interaction between sugarcane-derived polyphenols and pancreatic lipase.

Nevertheless, several limitations of the present study should be acknowledged. The inhibitory concentrations required in vitro were relatively high, and the IC_50_ values of crude extracts reflect total extract mass rather than the effective concentration of individual bioactive molecules. In addition, the bioavailability, metabolic stability, and intestinal fate of these polyphenols were not addressed, which may substantially influence their physiological relevance. Therefore, the current findings should be interpreted as mechanistic and screening-level evidence rather than direct proof of in vivo efficacy.

Overall, this work contributes to a deeper understanding of the structure–activity relationships and inhibition mechanisms of sugarcane-derived polyphenols toward pancreatic lipase. While the results suggest that sugarcane byproducts, particularly peel, represent a promising source of bioactive compounds, further validation using cellular and in vivo models is required before practical applications in nutrition or health-related contexts can be considered.

## Figures and Tables

**Table 1:** Total phenolic and total flavonoid contents in different sugarcane fractions.

Sample	Total Phenolic Content (mg GAE/g DM)	Total Flavonoid Content (mg RE/100 g DW)
SP	13.52 ± 0.87 ^a^	2074.65 ± 224.72 ^a^
SJ	4.89 ± 0.39 ^b^	560.79 ± 90.11 ^b^
SB	1.72 ± 0.13 ^c^	77.27 ± 38.06 ^c^

Note: Different superscript letters within the same column indicate significant differences (*p* < 0.05); SP: sugarcane peel; SJ: sugarcane juice; SB: sugarcane bagasse; DM, dry matter; DW, dry weight.

**Table 2:** Distribution and abundance of phenolic and flavonoid compounds in different sugarcane fractions.

Compound	SP (μg/g)	SJ (μg/g)	SB (μg/g)
**Phenolic Acids**			
Gallic Acid	97.12	26.21	5.70
Protocatechuic Acid	22.33	2.41	0.58
Chlorogenic Acid	87.98	-	-
Caffeic Acid	8.62	2.47	1.28
Ferulic Acid	128.22	-	-
Sinapic Acid	-	10.03	1.30
**Flavonoids**			
Schaftoside	-	5.78	0.67

**Table 3:** Comparison of IC_50_ Values of Different Mixtures and Monomers.

Category	Sample	IC_50_ (mg/mL)
Mixtures	SP	31.56 ± 1.62 ^c^
	SJ	55.86 ± 2.10 ^b^
	SB	65.31 ± 3.19 ^a^
Monomers	GA	1.04 ± 0.11 ^b^
	ChA	0.31 ± 0.05 ^c^
	CA	0.87 ± 0.03 ^b^
	FA	0.18 ± 0.04 ^c^
	SF	0.13 ± 0.02 ^c^
	SA	1.33 ± 0.10 ^a^

Note: Different superscript letters indicate significant differences (*p* < 0.05). SP, sugarcane peel; SJ, sugarcane juice; SB, sugarcane bagasse; GA, gallic acid; ChA, chlorogenic acid; CA, caffeic acid; FA, ferulic acid; SF, schaftoside; SA, sinapic acid.

**Table 4:** Kinetic parameters for pancreatic lipase inhibition by six sugarcane polyphenols.

	Concentration (mg/mL)	$V_{m}$ $/V_{m}^{app}$ $\left(\times 10^{3}mg/(mL\cdot min)\right)$	$K_{m}$$/K_{m}^{app}$ (mg/mL)	$K_{i}$ (mg/mL)	$K_{is}$ (mg/mL)
GA	0.0	0.0400	0.332	-	-
	0.4	0.0395	0.555	0.595	32.23
	1.2	0.0375	1.008	0.536	17.74
ChA	0.0	0.0318	0.118	-	-
	0.5	0.0320	0.151	1.859	-
	1.0	0.0319	0.253	0.888	-
CA	0.0	0.1163	2.584	-	-
	0.6	0.1028	2.690	3.374	4.507
	1.0	0.0736	2.972	1.225	1.727
FA	0.0	0.0355	0.153	-	-
	0.2	0.0345	0.183	0.897	6.972
	0.7	0.0322	0.278	0.700	6.932
SF	0.0	0.0383	0.0312	-	-
	0.2	0.0320	0.691	0.121	1.036
	0.4	0.0219	1.298	0.064	0.535
SA	0.0	0.0742	0.768	-	-
	0.3	0.0683	1.008	0.704	3.485
	1.0	0.0649	1.113	1.520	6.944

Note: GA: gallic acid; ChA: chlorogenic acid; CA: caffeic acid; FA: ferulic acid; SF: schaftoside; SA: sinapic acid, “-” indicates not applicable.

**Table 5:** Secondary structure proportions of pancreatic lipase treated with sugarcane polyphenols.

Sample	Secondary Structure Area Proportion (%)			
	β-Sheet	Random Coil	α-Helix	β-Turn
Pancreatic Lipas	50.34 ± 2.59	22.03 ± 4.79	11.52 ± 2.29	16.11 ± 0.45
Gallic Acid	40.63 ± 3.45	26.13 ± 5.69	12.35 ± 3.74	20.89 ± 5.99
Chlorogenic Acid	45.73 ± 2.57	20.44 ± 5.04	8.27 ± 1.24	25.56 ± 4.52
Caffeic Acid	43.18 ± 5.44	24.39 ± 5.42	8.88 ± 1.49	23.55 ± 1.32
Ferulic Acid	46.49 ± 3.48	20.17 ± 4.36	8.65 ± 1.00	24.70 ± 5.91
Schaftoside	41.16 ± 4.93	19.57 ± 4.63	23.63 ± 1.65	15.64 ± 1.35
Sinapic Acid	51.20 ± 6.31	16.50 ± 3.79	14.86 ± 1.61	17.45 ± 4.27

**Table 6:** Fluorescence quenching parameters of PL–polyphenol systems at different temperatures.

Polyphenol	T (K)	K_sv_ (mL/mg)	R^a^	K_q_ (×10^9^ mL/(mg·s))	K_a_ (mL/mg)	*n*	R^b^
Gallic Acid	298	4.71	0.98	1.57	2.86	1.02	0.98
	304	5.61	0.97	1.87	2.15	0.97	0.98
	310	7.11	0.97	2.37	3.52	1.05	0.99
Chlorogenic Acid	298	78.16	0.99	26.05	45.3	1.18	0.96
	304	90.09	0.96	30.03	38.62	1.09	0.95
	310	98.25	0.96	32.75	89.13	1.23	0.94
Caffeic Acid	298	72.54	0.95	24.18	28.47	0.95	0.93
	304	85.24	0.94	28.41	32.15	1.03	0.92
	310	105.34	0.94	35.11	41.69	1.11	0.95
Ferulic Acid	298	31.96	0.92	10.65	12.7	1.08	0.94
	304	55.49	0.98	18.5	21.54	1.15	0.97
	310	66.69	0.96	22.23	25.12	1.01	0.96
Schaftoside	298	47.38	0.96	15.79	18.42	0.93	0.92
	304	38.15	0.92	12.72	15.68	0.89	0.93
	310	26.14	0.98	8.71	8.76	1.07	0.94
Sinapic Acid	298	42.12	0.95	14.04	16.23	1.04	0.95
	304	24.69	0.96	8.23	11.35	0.98	0.92
	310	24.42	0.94	8.14	10.91	1.02	0.94

Note: Ksv, Stern–Volmer quenching constant; K_q_, quenching rate constant; K_a_, binding constant; n, number of binding sites; R^a^, correlation coefficient of the Stern–Volmer plot; R^b^, correlation coefficient of the double-logarithmic plot.

**Table 7:** Binding energies of sugarcane polyphenols with pancreatic lipase obtained from molecular docking.

Phenolic Compounds	GA	ChA	CA	FA	SF	SA	pNPL
Binding energy with lipase (kj·mol^−1^)	−6.0	−8.7	−6.6	−6.7	−9.0	−7.0	−7.7

Note: GA: gallic acid; ChA: chlorogenic acid; CA: caffeic acid; FA: ferulic acid; SF: schaftoside; SA: sinapic acid.

## Data Availability

The original contributions presented in this study are included in the article/Supplementary Materials. Further inquiries can be directed to the corresponding author.

## Supplementary Materials

The following supporting information can be downloaded at: https://www.mdpi.com/article/10.3390/foods15091480/s1, Figure S1: Reversibility plots for the inhibition of pancreatic lipase (PL) by six sugarcane polyphenols. (a) gallic acid (GA), (b) chlorogenic acid (ChA), (c) caffeic acid (CA), (d) ferulic acid (FA), (e) schaftoside (SF), and (f) sinapic acid (SA); Figure S2: Lineweaver–Burk double-reciprocal plots showing the inhibition of pancreatic lipase (PL) activity by six sugarcane polyphenols at different concentrations. (a) gallic acid (GA), (b) chlorogenic acid (ChA), (c) caffeic acid (CA), (d) ferulic acid (FA), (e) schaftoside (SF), and (f) sinapic acid (SA).

## References

[1] Wang Y., Zhao L., Gao L., Pan A., Xue H. (2021). Health policy and public health implications of obesity in China. Lancet Diabetes Endocrinol..

[2] Sun X., Yan A.F., Shi Z., Zhao B., Yan N., Li K., Gao L., Xue H., Peng W., Cheskin L.J. (2022). Health consequences of obesity and projected future obesity health burden in China. Obesity.

[3] Gaur P., Khan F., Shanker K. (2023). Potential lipase inhibitor from underutilized part of *Andrographis paniculata*: Targeted isolation and mechanism of inhibition. Ind. Crops Prod..

[4] Harp J.B. (1999). Orlistat for the long-term treatment of obesity. Drugs Today.

[5] Henness S., Perry C.M. (2006). Orlistat: A review of its use in the management of obesity. Drugs.

[6] Noorolahi Z., Sahari M.A., Barzegar M., Ahmadi Gavlighi H. (2020). Tannin fraction of pistachio green hull extract with pancreatic lipase inhibitory and antioxidant activity. J. Food Biochem..

[7] Chauhan P., Kumar R.R., Mendiratta S.K., Talukder S., Gangwar M., Sakunde D.T., Meshram S.K. (2021). In-vitro functional efficacy of extracts from *Phyllanthus emblica*, *Eucalyptus globulus*, *Tinospora cordifolia* as pancreatic lipase inhibitor and source of anti-oxidant in goat meat nuggets. Food Chem..

[8] Moliner C., Núñez S., Cásedas G., Valero M.S., Dias M.I., Barros L., López V., Gómez-Rincón C. (2023). Flowers of *Allium cepa* L. as Nutraceuticals: Phenolic Composition and Anti-Obesity and Antioxidant Effects in Caenorhabditis elegans. Antioxidants.

[9] Cao Q., Mei S., Mehmood A., Sun Y., Chen X. (2024). Inhibition of pancreatic lipase by coffee leaves-derived polyphenols: A mechanistic study. Food Chem..

[10] Deseo M.A., Elkins A., Rochfort S., Kitchen B. (2020). Antioxidant activity and polyphenol composition of sugarcane molasses extract. Food Chem..

[11] Zheng R., Su S., Zhou H., Yan H., Ye J., Zhao Z., You L., Fu X. (2017). Antioxidant/antihyperglycemic activity of phenolics from sugarcane (*Saccharum officinarum* L.) bagasse and identification by UHPLC-HR-TOFMS. Ind. Crops Prod..

[12] Zhuo G., Xiong F., Ping-Ping W., Chin-Ping T., Chun C. (2025). Ultrasonic collaborative pulse extraction of sugarcane polyphenol with good antiaging and α-glucosidase inhibitory activity. Int. J. Biol. Macromol..

[13] Galić L., Lončarić Z., Lisjak M. (2025). A Review of Phenolic Compounds: From Biosynthesis and Ecological Roles to Human Health and Nutrition. Phyton-Int. J. Exp. Bot..

[14] Oliveira A.L.S., Carvalho M.J., Oliveira D.L., Costa E., Pintado M., Madureira A.R. (2022). Sugarcane Straw Polyphenols as Potential Food and Nutraceutical Ingredient. Foods.

[15] Wen Y., Zeng X., Tan H., Liu B. (2023). Optimization of extraction process of total flavonoids from *Cortex Lycii* and its biological activities. Biomass Convers. Biorefin..

[16] Buţerchi I., Ciurlă L., Enache I.M., Patraş A., Teliban G.C., Irimia L.M. (2025). Valorisation of beetroot peel for the development of nutrient-enriched dehydrated apple snacks. Foods.

[17] Jia Z., Tang M., Wu J. (1999). The determination of flavonoid contents in mulberry and their scavenging effects on superoxide radicals. Food Chem..

[18] Iwata K., Wu Q., Ferdousi F., Sasaki K., Tominaga K., Uchida H., Arai Y., Szele F.G., Isoda H. (2020). Sugarcane (*Saccharum officinarum* L.) Top Extract Ameliorates Cognitive Decline in Senescence Model SAMP8 Mice: Modulation of Neural Development and Energy Metabolism. Front. Cell Dev. Biol..

[19] Jaradat N., Hawash M., Dass G. (2021). Phytochemical analysis, in-vitro anti-proliferative, anti-oxidant, anti-diabetic, and anti-obesity activities of *Rumex rothschildianus* Aarons. extracts. BMC Complement. Med. Ther..

[20] Rahim A.T.M.A., Takahashi Y., Yamaki K. (2015). Mode of pancreatic lipase inhibition activity in vitro by some flavonoids and non-flavonoid polyphenols. Food Res. Int..

[21] Li S., Pan J., Hu X., Zhang Y., Gong D., Zhang G. (2020). Kaempferol inhibits the activity of pancreatic lipase and its synergistic effect with orlistat. J. Funct. Foods.

[22] Jiang W., Huang Q., Meng X., Rehman R.U., Qian K., Yang X., Wang H. (2023). Ursolic acid inhibited cholesterol esterase and pancreatic lipase activities and decreased micellar cholesterol solubility in vitro. J. Food Biochem..

[23] Feng S., Luo Z., Zhang Y., Zhong Z., Lu B., Phy H. (2014). Ursolic acid inhibited cholesterol esterase and pancreatic lipase activities and decreased micellar ctochemical contents and antioxidant capacities of different parts of two sugarcane (*Saccharum officinarum* L.) cultivars. Food Chem..

[24] Ni Y., Chen H., Liu D., Zeng L., Chen P., Liu C. (2021). Discovery of genes involved in anthocyanin biosynthesis from the rind and pith of three sugarcane varieties using integrated metabolic profiling and RNA-seq analysis. BMC Plant Biol..

[25] Lee E.M., Lee S.S., Chung B.Y., Cho J.Y., Lee I.C., Ahn S.R., Jang S.J., Kim T.H. (2010). Pancreatic Lipase Inhibition by C-Glycosidic Flavones Isolated from *Eremochloa ophiuroides*. Molecules.

[26] Jamai K., Daoudi N.E., Elrherabi A., Bnouham M. (2023). Medicinal Plants and Natural Products to Treat Obesity through Inhibiting Pancreatic Lipase: A Review (2020–2022). Lett. Drug Des. Discov..

[27] He X., Chen L., Pu Y., Wang H., Cao J., Jiang W. (2023). Fruit and vegetable polyphenols as natural bioactive inhibitors of pancreatic lipase and cholesterol esterase: Inhibition mechanisms, polyphenol influences, application challenges. Food Biosci..

[28] Hu B., Cui F., Yin F., Zeng X., Sun Y., Li Y. (2015). Caffeoylquinic acids competitively inhibit pancreatic lipase through binding to the catalytic triad. Int. J. Biol. Macromol..

[29] Eydoux C., Spinelli S., Davis T.L., Walker J.R., Seitova A., Dhe-Paganon S., De Caro A., Cambillau C., Carrière F. (2008). Structure of Human Pancreatic Lipase-Related Protein 2 with the Lid in an Open Conformation. Biochemistry.

[30] He X.Q., Zou H.D., Liu Y., Chen X.J., Atanasov A.G., Wang X.L., Xia Y., Ng S.B., Matin M., Wu D.T. (2024). Discovery of Curcuminoids as Pancreatic Lipase Inhibitors from Medicine-and-Food Homology Plants. Nutrients.

[31] Cao Q., Huang Y., Zhu Q.F., Song M., Xiong S., Manyande A., Du H. (2020). The mechanism of chlorogenic acid inhibits lipid oxidation: An investigation using multi-spectroscopic methods and molecular docking. Food Chem..

[32] Shen H., Wang J., Ao J., Ye L., Shi Y., Liu Y., Li M., Luo A. (2023). The inhibitory mechanism of pentacyclic triterpenoid acids on pancreatic lipase and cholesterol esterase. Food Biosci..

[33] Park J.Y., Kim C.S., Park K.M. (2019). Inhibitory characteristics of flavonol-3-O-glycosides from *Polygonum aviculare* L. (common knotgrass) against porcine pancreatic lipase. Sci. Rep..

[34] Martinez-Gonzalez A.I., Alvarez-Parrilla E., Díaz-Sánchez Á.G., de la Rosa L.A., Núñez-Gastélum J.A., Vazquez-Flores A.A., Gonzalez-Aguilar G.A. (2017). In Vitro Inhibition of Pancreatic Lipase by Polyphenols: A Kinetic, Fluorescence Spectroscopy and Molecular Docking Study. Food Technol. Biotechnol..

[35] Sun R., Gao J., Peng J., Lin Z., Wang Y., Wang J., Wei M., Lin Z., Tan J., Dai W. (2026). Characterization and mechanistic study on pancreatic lipase inhibitory effect of teas and their bioactive compounds. Food Chem..

[36] Du X., Bai M., Huang Y., Jiang Z., Chen F., Ni H., Li Q. (2018). Inhibitory effect of astaxanthin on pancreatic lipase with inhibition kinetics integrating molecular docking simulation. J. Funct. Foods.

[37] Huang X., Zhu J., Wang L., Jing H., Ma C., Kou X., Wang H. (2020). Inhibitory mechanisms and interaction of tangeretin, 5-demethyltangeretin, nobiletin, and 5-demethylnobiletin from citrus peels on pancreatic lipase: Kinetics, spectroscopies, and molecular dynamics simulation. Int. J. Biol. Macromol..

