# Supplementary material for: Mechanistic Insights into Pancreatic Lipase Inhibition by Sugarcane Polyphenols: A Structural and Kinetic Study
Source: Foods. 2026 Apr 23;15(9):1480. doi: 10.3390/foods15091480 (PMC13164236; doi:10.3390/foods15091480)
Supplement: Supplementary file 1 [file foods-15-01480-s001.zip › foods-4145947-supplementary.pdf]

Supplementary Information

Mechanistic Insights Into Pancreatic Lipase Inhibition by Sugarcane Polyphenols: A Structural and Kinetic Study

Qiyan Liu [1], Ping-Ping Wang [2], Xiong Fu [1,3,4] and Chun Chen [1,3,4,*]

[1] School of Food Science and Engineering, South China University of Technology, Guangzhou 510640, China

[2] School of Chemical Engineering and Light Industry, Guangdong University of Technology, Guangzhou 510006, China

[3] Zhuhai Institute of Modern Industrial Innovation, South China University of Technology, Zhuhai 519175, China

[4] Overseas Expertise Introduction Center for Discipline Innovation of Food Nutrition and Human Health (111 Center), Guangzhou 510640, China

* Correspondence: chenc@scut.edu.cn

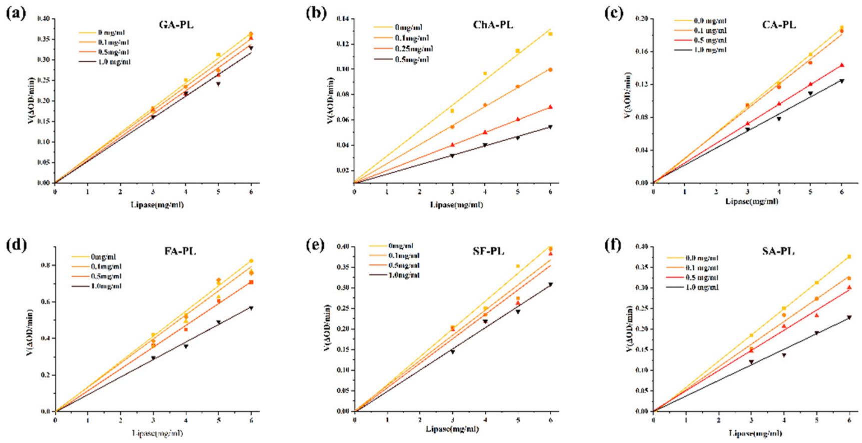

Figure S1. Reversibility plots for the inhibition of pancreatic lipase (PL) by six sugarcane polyphenols. (**a**) Gallic acid (GA), (**b**) chlorogenic acid (ChA), (**c**) caffeic acid (CA), (**d**) ferulic acid (FA), (**e**) schaftoside (SF), and (**f**) sinapic acid (SA). The plots illustrate the relationship between the reaction rate and PL concentration in the presence of varying concentrations of polyphenols. All regression lines pass through the origin, indicating that the inhibition of PL by these monomers is reversible.

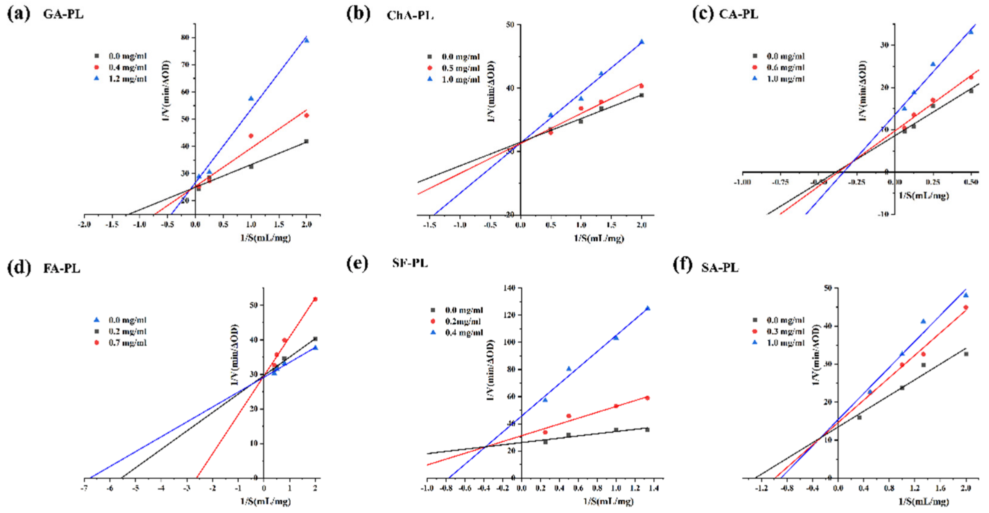

Figure S2. Lineweaver–Burk double-reciprocal plots showing the inhibition of pancreatic lipase (PL) activity by six sugarcane polyphenols at different concentrations. (**a**) Gallic acid (GA), (**b**) chlorogenic acid (ChA), (**c**) caffeic acid (CA), (**d**) ferulic acid (FA), (**e**) schaftoside (SF), and (**f**) sinapic acid (SA). Reactions were performed using p-nitrophenyl laurate (pNPL) as the substrate, and initial rates (V) were measured under varying substrate and polyphenol concentrations; 1/V was plotted against 1/[S].
